# An unusual urethral foreign body “AA battery” associated with urethral stenosis: A case report

**DOI:** 10.1016/j.eucr.2021.101921

**Published:** 2021-10-28

**Authors:** Jalil Hosseini, Saeid Abouei, Ali Mohammad Mirjalili

**Affiliations:** a Men's Health and Reproductive Health Research Center, Shahid Beheshti University of Medical Sciences, Tehran, Iran; b Student Research Committee, Faculty of Medicine, Shahid Sadoughi University of Medical Sciences, Yazd, Iran

**Keywords:** Urethra, Foreign body, Self-insertion, Urethroplasty

## Abstract

Urethral foreign bodies are rare with a variety of causes, including psychiatric illness, autoeroticism, intoxication, and perceived contraception, we present a 49-yr-old man who went to the emergency ward 9-month ago inserting an AA battery in his urethra. The battery was removed without surgery after 24 hours. The patient was then referred to the hospital after 5 months with complaints of obstructive urinary symptoms such as decreased urinary caliber, diagnosed with penile urethral stricture, and undergoes dilatation of the urethra. Usually, in the case of urethral stricture, the initial steps including urethral dilation and internal urethrotomy can be used.

## 1 Introduction

Urethral foreign bodies are relatively rare. Some of the different causes of this condition are psychiatric illness, autoeroticism, intoxication, and perceived contraception.^1^ The patient can present with dysuria, blood in the urethra, sepsis or even no symptom. Prolonged stay of the foreign body in the urethra can lead to a urethral stricture.^2^ Foreign bodies are diverse and depending on their location, there are several techniques for extracting. However, in the case of urethral stricture, grafts can be used for repairing.

## 2 Case presentation

**Fig. 1:**
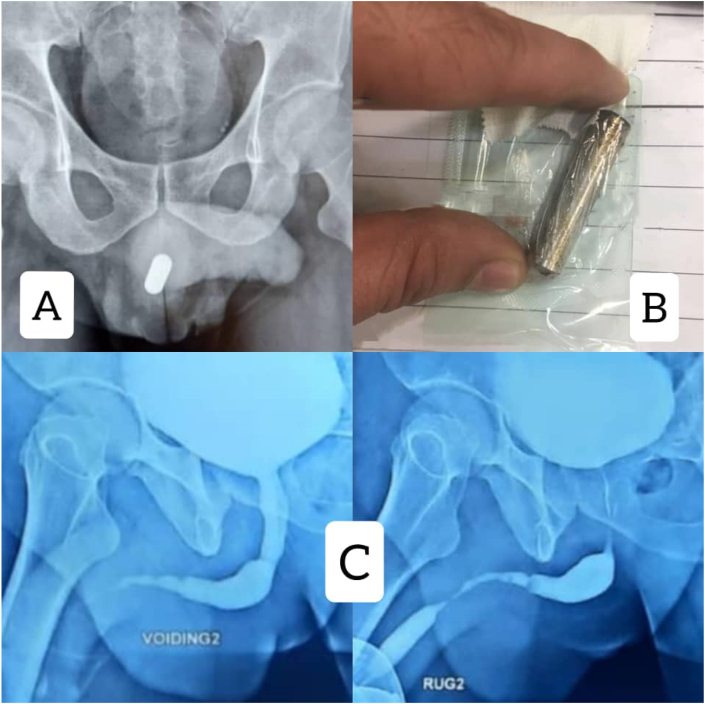
**A**: initial X-Ray in emergency ward**. B:** The battery was removed in emergency ward. **C:** VCUG&RUG before operation shows Penile Urethral stricture 6–8 cm in length, with normal bladder.

**Fig. 2:**
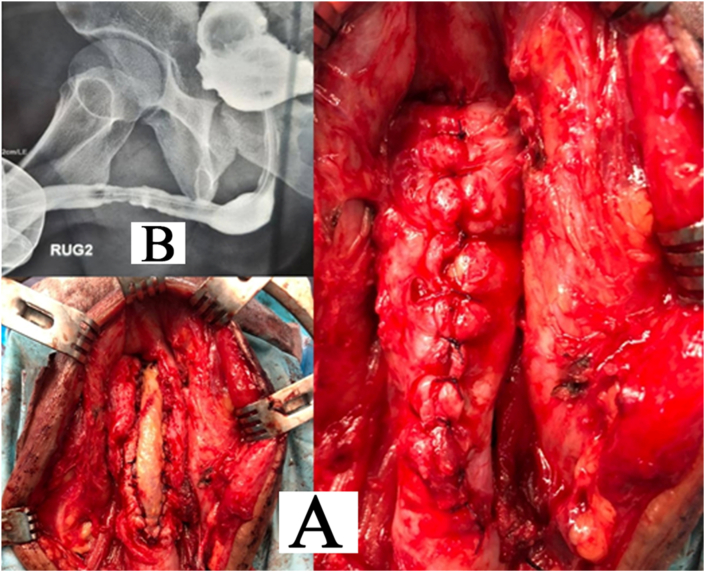
**A:** buccal ventral Onlay Urethroplasty and Spongioplasty**. B:** post operation pre catheter RUG.

The patient was a 49-year-old man who was referred to the emergency ward 24 hours after inserting an AA battery into his urethra, 9 months ago. The patient underwent an x-ray and the battery was seen in his urethra (Fig. 1 A&B). The battery was then removed without surgery. He then was referred to the hospital after 5 months with a complaint of obstructive urinary symptoms such as decreased urinary caliber, then was diagnosed with penile urethra stricture and underwent urethra dilatation. He had not had any surgery, cystostomy, or CIC (clean intermittent catheterization) during this period. The patient complained of recurrence of obstructive and irritating urinary symptoms including dysuria, decreased urinary force, decreased urinary caliber but no hematuria. The patient had not any history of underlying diseases such as mental illness and the use of neuropsychiatric drugs. There was a 6–8 cm stricture in the bulbopenile urethra which is shown in Retrograde Urethrogarphy (RUG) (Fig. 1C). He was prepared for oral mucosa graft Urethroplasty with the rigid 17 F cystoscope, it was observed that the urethral stricture was situated at 7 cm distal from the meatus, through which the cystoscope could no longer pass. The urethra was revealed with perineum incision and bulbospongiosum muscle was incised. The stricture had 8 cm long. After harvesting and trimming of buccal graft, it was used as the ventral Onlay and sutured with Vicryl 4–0 on Silastic catheter 18F (Fig. 2A). Everything was normal in the hospital and three weeks later, he presented for RUG through a pre-catheter that showed no leakage of radiocontrast agent (Fig. 2B). Foley catheter and cystostomy were removed. The patient was satisfied with his voiding at a 6-month follow-up.

## 3 Discussion

There are several reasons for inserting a foreign body into the penis, including psychiatric illness, autoeroticism, intoxication, and perceived contraception.^1^^,^^2^ Several examples of these cases have been observed in both men and women, which is more common in men and is also present at all ages.^3^ The foreign body can be various objects such as materials, wires, toothbrushs, batteries, etc. Visiting a doctor is usually unpleasant and the patient may be ashamed of it, but eventually, they visit the doctor due to urinary symptoms including dysuria, pain, or hematuria. But, in some cases, there are no symptoms, so delay in visiting the doctor can lead to a complicated infection or Fournier gangrene.^2^^,^^4^ These cases are usually easy to diagnose and can be understood based on the clinical history and the touch of a foreign body from the penis. Urinary Radiographs can also be helpful, and if the foreign body is not radiopaque, a pelvic scan or cystoscopy may be needed. There are several methods for removing a foreign body including non-surgical means (using basket or forceps), endoscopy, and finally open surgery; the first and second procedures are performed at first and the surgery remains the last option. The location, shape, and duration before visiting a doctor are important components in determining the procedure. If the foreign body remains in the urethra for a long time, the patient may experience consequences such as erectile dysfunction, urethral diverticulum, and urethral stricture.^5^

In this case, the presence of a battery in the urethra for 24 hours caused severe and progressive damage to the urethra and corpus spongiosum. Internal Urethrotomy intensified fibrosis and increased the length of stenosis. The urethra had severed adhesions to surrounding tissues during Urethroplasty and severe fibrosis of the corpus spongiosum was evident. Due to the 8 -cm pinpoint stricture in the bulbopenile urethra and no possibility of passing the rigid or flexible cystoscope, a graft was needed. Two of the most popular grafts are PSG and BMG which are used for urethral stricture.

## 4 Conclusion

In dealing with a patient having a foreign body in the urethra, his history and clinical examination can be a big step in diagnosing and choosing the type of treatment to be practiced. Moreover, radiography is needed for the exact shape and position of the foreign body although RUG and VUCG can also be used to see into the duct. Due to the patients being inconvenient to see the doctor, they may return late and the foreign body remains in the duct for some time, which in itself causes many problems, including urethral strictures and urinary tract infections. If urethral stricture occurs, the initial steps can be used, including internal Urethrotomy, and finally, by failing the above methods, Urthroplasty can be performed for the patient.

## Consent for publication

Written informed consent was obtained from the patient for publication of this case report and any accompanying images. A copy of this written consent is available for review by the editor of the journal.

## Competing interests & funding

No sources of funding have to be declared.

## Abbreviations

CIC: Clean Intermittent Catheterization
RUG: Retrograde Urethroplasty
BMG: Buccal mucosal graft
PSG: penile skin graft
VCUG: Voiding Cystourethrogram
